# Path probability selection in nature and path integral

**DOI:** 10.1038/s41598-022-20235-2

**Published:** 2022-11-09

**Authors:** Chao Wang, Min-Lan Li, Rui-Wu Wang

**Affiliations:** 1 School of Ecology and Environment, Northwestern Polytechnical University, Xi’an, 710072 Shaanxi People’s Republic of China; 2 School of Mathematics and Statistics, Northwestern Polytechnical University, Xi’an, 710072 Shaanxi People’s Republic of China

**Keywords:** Evolutionary theory, Nonlinear phenomena

## Abstract

Understanding of any biological evolutions, such as speciation, adaptation behavior and biodiversity pattern, is based on a fundamental concept of fitness, in which natural selection implies the improvement and progress of fitness in either direct/indirect benefit or genetic transmission to the next generation. However, this basic idea of biological evolution, which is mathematically described by Price equation or its related models, has not fully considered feedback effects from the environment or other generations. They lost the global dynamics of the evolutions consequently. Drawing on the idea of modern physics, we introduce the path integral by iterating the Price equation step by step to characterize the evolutionary path in which the stationary fitness is replaced by the path probability. The evolutionary selection therefore will depend on path probability instead of fitness advantage. In such a framework of the evolutionary path, the intermediate process of evolution is not always pointing to the fitness-maximizing equilibrium and multiple evolutionary paths could thus coexist without fitness advantage discrimination. This mechanism could potentially explain evolutionary strategies with the diversified fitness (e.g., coexistence of altruism and selfishness) and thus species diversity.

## Introduction

**Figure 1:**
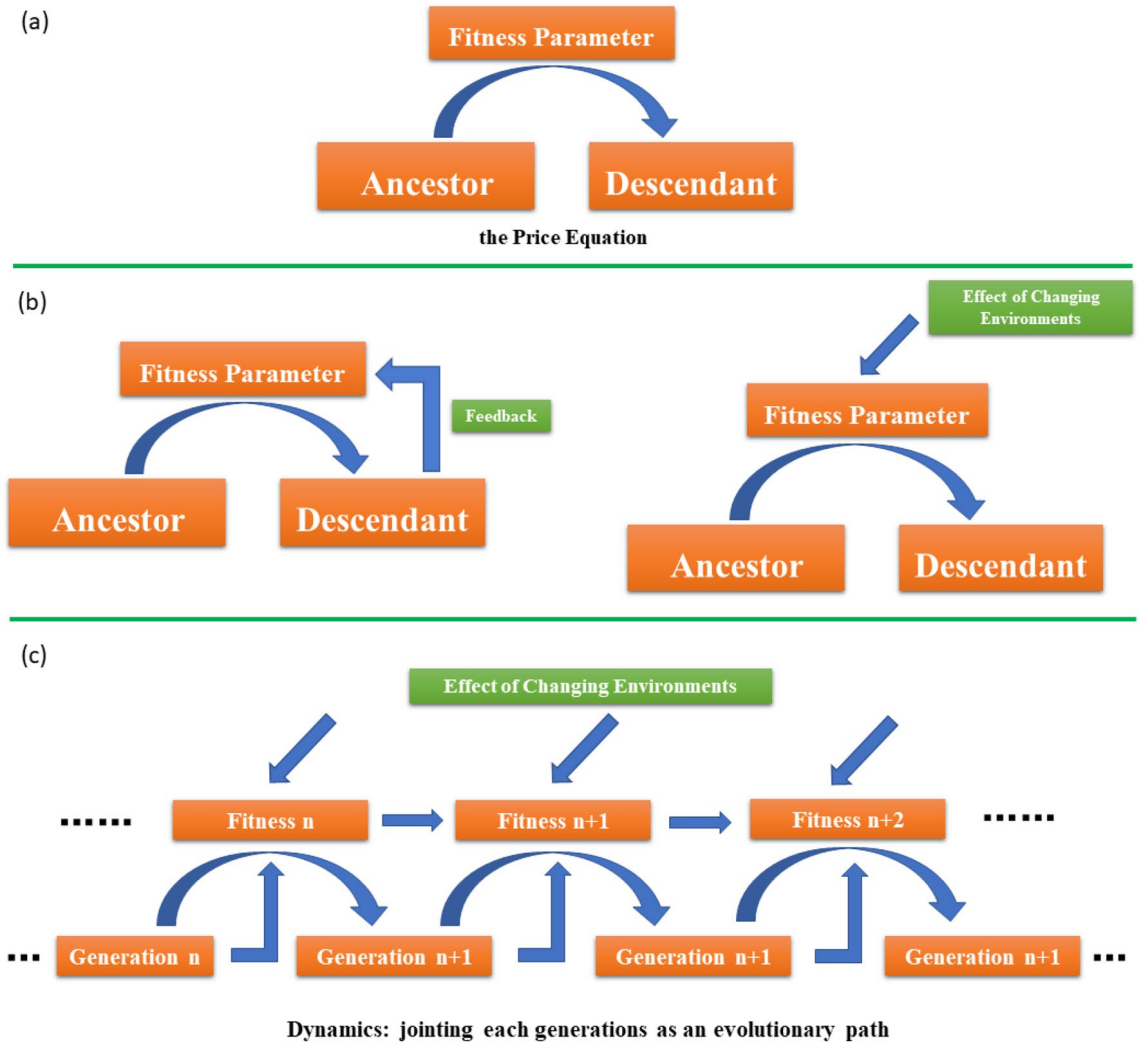
The Price equation and the evolutionary trajectory. (**a**) The Price equation describes evolutionary change between generations through the covariance of a character with its fitness. (**b**) The fitness of a generation might receive feedback originated from the change in quantity and character of the last generation or other more generations. The population distribution might also be affected by their living environments. (**c**) The whole evolutionary trajectory should be successive by jointing each generation step by step.

Evolution through natural selection is often understood to imply improvement and progress in fitness advantage which could be either direct/indirect fitness or genetic transmission to the next generation^1–5^. A trait, if it could increase the individual’s fitness, will spread in the population and the average fitness of the population will therefore increase over time^2,4,6^. Such a metaphor of steady ascent on fitness landscape suggests some solid ground over which the population moves^7–9^. This scenario is described by Price equation mathematically, in which the character change is equal to the covariance between the character and its fitness (Fig. 1a)^3–5,10^.

However, these traditional theories are resorted to the stationary fitness assumption and neglects some important evolutionary dynamics: although the environment selects the adaptations, these adaptations can shape the environment^9,11–14^. As Nowak and Sigmund metaphorized that selective advantage of a given tree height will also depend on the heights of neighboring trees, the fitness of the individuals will not just be affected by the phenotypic distributions of its own population, but also be greatly connected with the phenotype change of other species populations^9^, as well as the interaction variation between ecological and biological factors^15–19^. When the fitness of a biological organism varies over either time or spatial scale, which might be resulted from both intrinsic genotype/phenotype uncertainty and environmental fluctuations, a uniform fitness throughout the whole evolution processes therefore might not be proper to describe evolutionary advantages of an organism^20^. The instantaneous fitness at a moment of the evolution, instead of uniform fitness as a stationary fitness, will be more appreciated and credible to describe the evolutionary process of a biological organism^21–23^.

Instead of integrating frequency- or environment-dependence into population intrinsic fitness and concentrating the localized property near the equilibrium point (or steady state) (e.g., evolutionary game theory, adaptive dynamics, and population genetics)^20,24–27^, here we state dynamics of the evolutionary history with the instantaneity of the fitness and focus on the evolutionary trajectories^22,28–31^. We put forward the evolutionary path probability to characterize the evolutionary trajectories. In this manner, each evolution step recurs taking the feedback effect among generations into account in place of the scenarios of a population evolving through the fitness advantage. In present work, we draw on a mathematical approach based on path-integral technique, which was introduced by Feynman for quantum physics and has been proved to be a very powerful tool in various areas of physics, both computationally and conceptually^32^. The path integral method, which was raised from Wiener^33^ for Brownian motion, has found substantial success in applications in quantum mechanics and quantum field theory. In the 1940s Feynman^32^ formulated a space–time approach to quantum mechanics based on the path integral introduced by himself. Recent years have witnessed a majority efforts to develop a similar path integral formulation for the theory of the stochastic process. And such a formulation can supply a calculational technique that may be fruitful in dealing with nonlinear systems. Although general evaluation of the path integral is impossible for nonlinear systems, its definition as the limit of a multidimensional integral suggests powerful numerical methods^34–36^. Recently, Joshua has performed attempting for the evolutionary problem of the gene frequency in the population genetics, showing how it is possible to use path integral methods to express the transition probability by means of a functional integral over paths of the evolutionary process for the Wright-Fisher process^37^. We also present a typical example on account of evolutionary game for approximating the evolutionary trajectory to make the conception more practical.

## Mathematical background and the formulation of recursive evaluation

For sanity of our conceptions, we start from the simple Price’s equation and then derivate the evolutionary path probability. In general, fitness is interpreted as the expected reproductive success of a trait^3,5^. The reproductive success is estimated in relation to a reference population: they are the linear combination of allelic frequencies that best explains phenotypic variation in the trait^3,38^. The Price’s rule indicates that the evolutionary change in the average value of some traits, *z,* can be written as^3,5^ (Fig. 1a)

1 $$\overline{\omega }\Delta {\overline{z }}=Cov\left(z,\omega \right)+E(\omega\Delta z),$$

where the fitness, $\omega$, of a trait measures the number of its descendants at future time. The second term of the Price equation is sometimes added to account for non-Mendelian effects (e.g., meiotic drive) and random statistical deviations (e.g., genetic drift). To make the analysis more explicit, the second term is negligible without losing credibility in the following.

In the Price equation, the change in the average value of some property between two populations could be described. We consider a population (a set) in which each element is labeled by a label *i*. The label *i* may be used as any sort of property of trait ${z}_{i}$ in the set, such as allele, genotype, phenotype, group of individuals, and so on. The frequency of elements with index *i* is ${q}_{i}$. For its descendant, the corresponding frequencies ${q}_{i}^{^{\prime}}$ and the characters ${z}_{i}^{^{\prime}}$ have the trait with the label *i*. We use $\Delta {q}_{i}$ for frequency change associated with selection, and for property value change $\Delta {z}_{i}$=0 as we neglect the mutation. The total change $\Delta {\overline{z }}$ is thus

2 $$\begin{aligned}\Delta {\overline{z }}=\overline{{z }^{\mathrm{^{\prime}}}}-{\overline{z }}& =\sum {q}_{i}^{\mathrm{^{\prime}}}{z}_{i}^{\mathrm{^{\prime}}}-\sum {q}_{i}{z}_{i}\\ &=\sum {q}_{i}^{\mathrm{^{\prime}}}{(z}_{i}^{\mathrm{^{\prime}}}-{z}_{i})+\sum {q}_{i}^{\mathrm{^{\prime}}}{z}_{i}+\sum {q}_{i}{z}_{i}\\ &=\sum {q}_{i}^{\mathrm{^{\prime}}}\Delta {z}_{i}+\sum\Delta {q}_{i}{z}_{i}.\end{aligned}$$

By following the prior definition of fitness, we have

3 $$\Delta {q}_{i}={q}_{i}^{\mathrm{^{\prime}}}-{q}_{i}={q}_{i}\frac{\omega }{\overline{\omega }}-{q}_{i}={q}_{i}\left(\frac{\omega }{\overline{\omega }}-1\right)=\frac{Cov\left(z,\omega \right)}{\overline{\omega }}.$$

This expression corresponds to the total change caused by changes in frequency. We call this the part caused by selection, because this is the part that arises directly from differential contribution by ancestors to the descendant population^5^. In this formular, the update information of fitness is not provided. In a stable fitness landscape or a short time, one can iterate the Price equation step by step with the fitness being regarded as a constant^38^. For the recursive evolutionary path, the fitness parameter $\omega$ of the evolutionary change between generations would receive feedback originated from the change in quantity and character of the last generation (and even other more generations), such as the evolution dynamic in the finite populations^9,20^. One should consider the nonlinear relationship (Fig. 1b)^27^. Also, the environmental impacts are included as a constant parameter for the fitness parameter in the traditional Price Equation, which would be liable to failure in long-term evolution (Fig. 1b). Instead of the fixed fitness, the fitness parameter dynamic also exists and the instantaneous fitness would offer a better way than the average fitness over time or stationary fitness^13^. We give the recurrence relation of the traits by substituting the instantaneous fitness ${\omega }^{(t)}=f[(q(t)]+\xi (t)$ instead of stationary fitness, where the first term comes from the feedback of population structure and $\xi \left(t\right)$ represents the impact of the environmental variation. The whole evolutionary process emerges from the recursive manner of the Price equation, which is a chain-like path (Fig. 1c).

**Figure 2:**
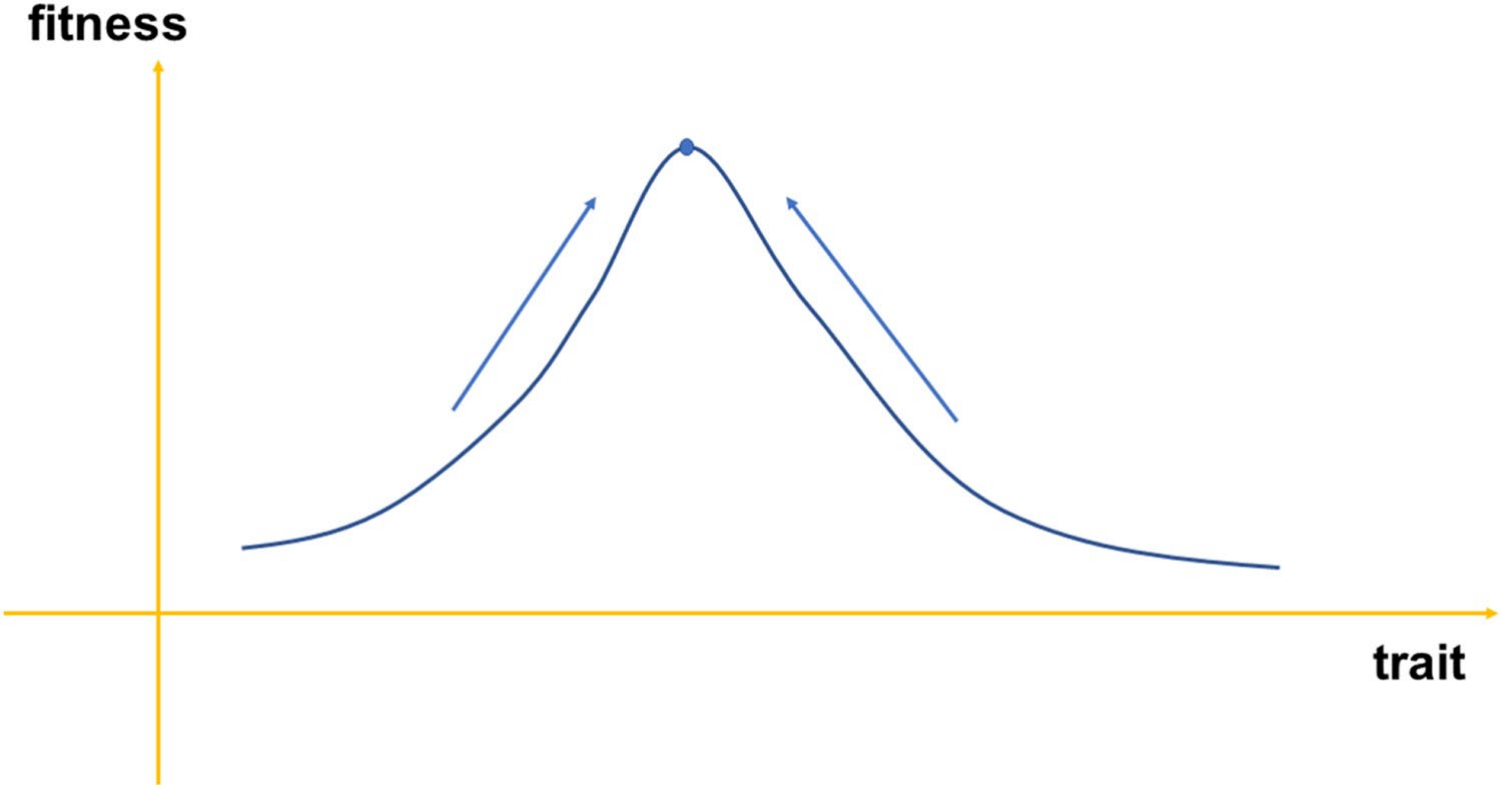
The evolutionary for the constant fitness distribution. In the traditional Price equation, where the fitness is constant, evolution always point to the equilibria. It is irrelevant to the traits. The evolution is only determined by the stationary fitness distributions and all the individual will evolve to position corresponding to the fitness maximum.

In the traditional Price equation, where the fitness is constant, at length evolution reach the equilibrium point $Cov\left(z,\omega \right)=0$. The evolution is determined by a stationary fitness distribution and the trait will always evolve to the fitness maximum point (shown in Fig. 2). At arbitrary time, the evolutionary direction is fixed toward the maximum point^2,4,5,29,30^. This evolutionary direction is irrelevant to the process*.* For a long evolutionary process and the fitness is frequency- dependent, the case changes. We assume that ${q}_{i}^{{(t}_{0})}$ is the frequencies in the initial state, and ${q}_{i}^{{(t}_{n})}$ is defined as the fraction of the descendant population at the generation *n* that is derived from members of the ancestral population that have the trait with the label *i*. If $i$=1, 2, ${q}_{1, 2}^{{(t}_{n})}$ is the fraction of the sequence descendants derived from entities with the phenotype $i$=1, 2 in the ancestors, therefore, *i* also represents a special evolution trajectory in the condition of neglecting random mutation. The fitness would rely on the frequency of the corresponding trait at last time with the recursion formula satisfied the Price equation:

4 $${q}_{i}^{{(t}_{n})}= {q}_{i}^{{t}_{n-1}}\left(\frac{{w}_{i}^{{(t}_{n})}}{{\overline{w} }^{{(t}_{n})}}\right).$$

${w}_{i}^{{(t}_{n})}({q}_{i}^{({t}_{n})})$, above equation can be recombined as

5 $${q}_{i}^{{(t}_{n})}= {q}_{i}^{{(t}_{0})}\prod_{k=0}^{n} \left (\frac{{w}_{i}^{{(t}_{k})}}{{\overline{w} }^{{t}_{k}}}\right)$$

In this expression, the frequency of the individual in the *i*-th evolution trajectory, ${q}_{i}^{{(t}_{0})}, {q}_{i}^{{(t}_{1})}, {q}_{i}^{{(t}_{2})}, \dots {q}_{i}^{{(t}_{n})}$ for discrete formula and ${q}_{i}^{{(t}_{n})}$ for continuous case, receive effects from feedback on the fitness, including its own and others’. The evolution direction is no longer fixed and would depend on the choice of the path *i*, i.e., the path dependent selection.

**Figure 3:**
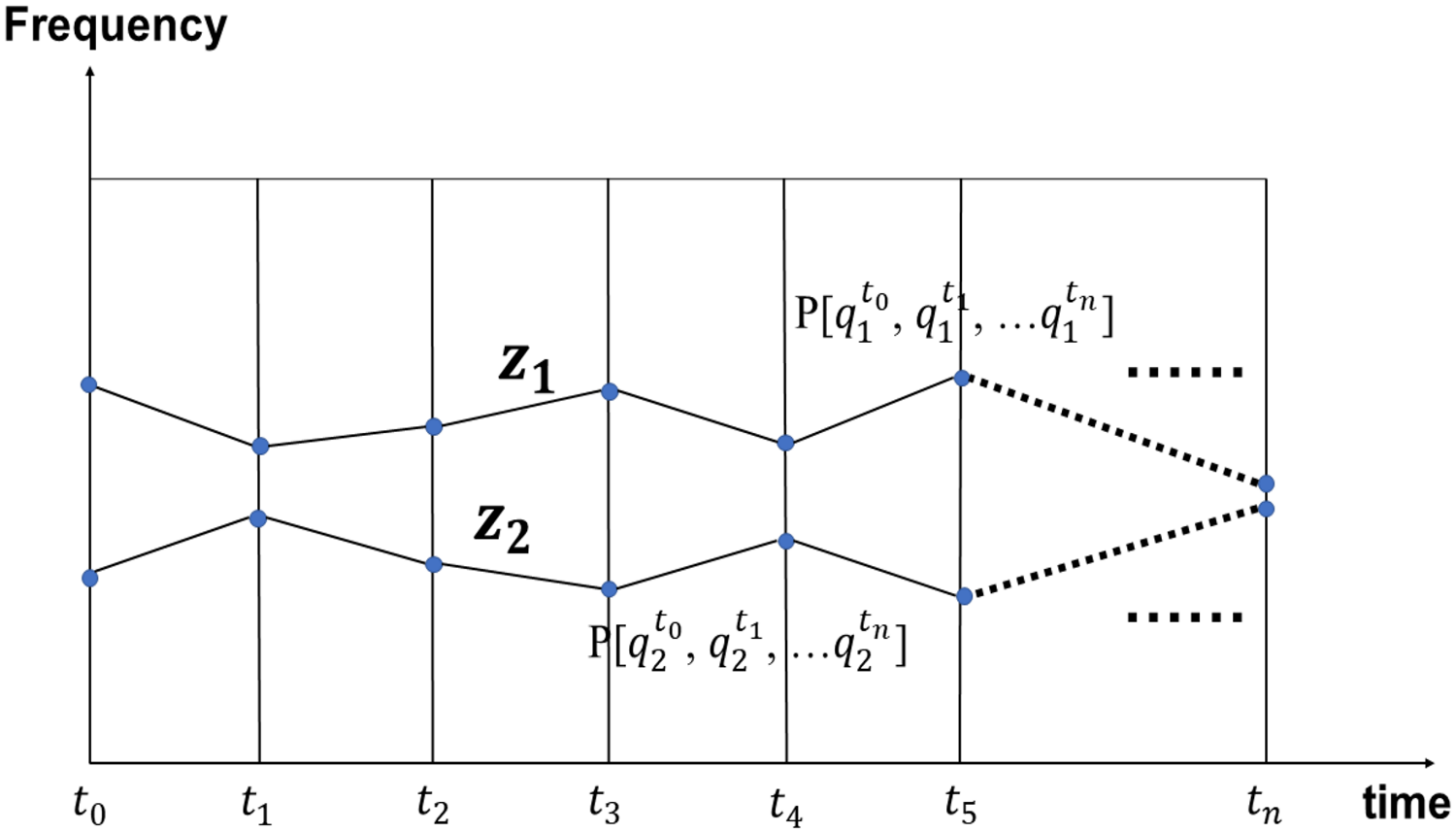
The evolutionary trajectories. There are two evolutionary pathways from time ${t}_{0}$ to time ${t}_{n}$, say path z_1_ and path z_2_. Each evolutionary path appears with the possibility of P[${q}_{1, 2}^{(t)}$] whose independent variable is a time dependent function [the evolutionary trajectories ${q}_{1, 2}^{(t)}$,]. For the discrete case, the distribution of the probability P[${q}_{1, 2}^{(t)}$] can be written as ${\mathrm{P}}_{\mathrm{path}}[{q}_{1, 2}^{{(t}_{0})}$, ${q}_{1, 2}^{{(t}_{1})}$, ${q}_{\mathrm{1,2}}^{{(t}_{2})}$, …${q}_{\mathrm{1,2}}^{{(t}_{n})}]$. The path distribution is satisfied ${q}_{1}^{(\mathrm{t})}+{q}_{2}^{(\mathrm{t})}$=1 at each time point $\mathrm{t}$.

Drawing on statistic physics, we introduce a conception of the probability distribution function (or rather functional) to identify the evolutionary path^32^. We sign this expanded generalized function distribution as ${\mathrm{P}}_{\mathrm{path}}$[${q}_{i}^{{(t}_{n})}$], whose independent variable is a time dependent function [the evolution trajectories ${q}_{i}^{{(t}_{n})}$]^32^. Then, each trajectory has an evolutionary path probability, P_path,_ as shown in Fig. 3 (for simplicity *i* = 1, 2). This evolutionary-path probability P_path_ [${q}_{i}^{{(t}_{0})}, \; {q}_{i}^{{(t}_{1})}, \; {q}_{i}^{{(t}_{2})}, \; \ldots {q}_{i}^{{(t}_{n})}$] can be thought of as similar to the probability function for a number of dependent variables ${q}_{i}^{{(t}_{0})}$, ${q}_{i}^{{(t}_{1})}$, ${q}_{i}^{{(t}_{2})}$, …, and ${q}_{i}^{{(t}_{n})}$, which is an infinite dimensional function. This probability distribution function can be used to infer the preference for evolutionary trajectory in the evolutionary process. A comprehensible mathematical detail of the evolutionary path probability given by the path integral is shown in the “Method”.

**Figure 4:**
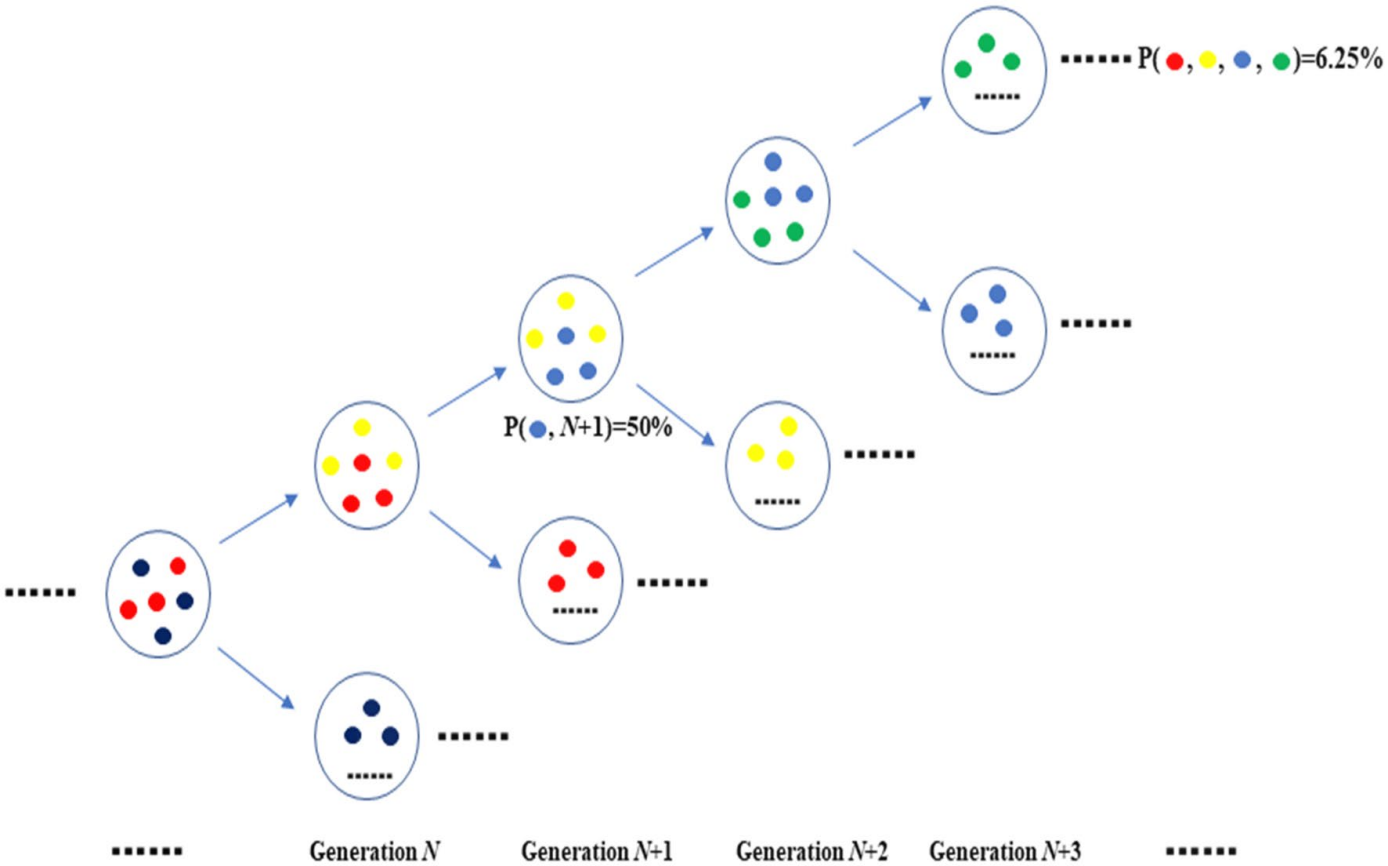
The probability distribution for strategies at a given time, P(${x}_{i}, t$) and the probability of the path of evolutionary trajectories, P[x(t)]. The traits are marked by colors. At the generation *N* + 1, the frequency of the blue type is 50% in the population. At each time step, there are two phenotypes and each one is selected by the probability of 0.5. If we consider the four-generation case, the trajectory (the sequence “red, yellow, bule, green”) with the frequency of the blue type being 50% in the generation *N* + 1 is 0.5 * 0.5 * 0.5 * 0.5 = 0.0625.

In fact, the population frequency ${q}_{i}$ and the evolutionary path probability ${\mathrm{P}}_{\mathrm{path}}$ are not consistent except in some extreme cases. At the time ${t}_{n}$, the individual adopts the phenotype *i* (Fig. 3) with the probability of ${q}_{i}^{({t}_{n})}$ ($\sum {q}_{i}^{({t}_{n})}$=1 at each time point ${t}_{n}$). By expanding to the distribution of pathway, one still could return to the trait distributions of ${q}_{i}^{{(t}_{n})}$ at arbitrarily evaluation time point, ${q}_{i}^{{(t}_{n})}=\sum_{i}{\mathrm{P}}_{\mathrm{path}}$. The mean value of the phenotypes at a given time point could be $\sum_{i}{z}_{i}(t)\sum_{i}{\mathrm{P}}_{\mathrm{path}}$ instead of $\sum_{i}{z}_{i}{q}_{i}$ in the Price equation, in which the path integral is instead by summation. For example, as shown in Fig. 4, the blue traits appear in *N* + *1* generation with 50% while the probability of the path with the blue traits in *N* + *1* generation is 6.25%. Only in one small step or a fitness stationary process, the phenotype strategy $i$ is fixed, and ${q}_{i}^{{(t}_{n})}={\mathrm{P}}_{\mathrm{path}} [{q}_{i}^{{(t}_{0})}, \; {q}_{i}^{{(t}_{1})}, \; {q}_{i}^{{(t}_{2})}, \; \ldots {q}_{i}^{{(t}_{n})}]$.

## An example

We will take a simulation of evolutionary game to show the path probability, ${\mathrm{P}}_{\mathrm{path}}$, and give a practical execution below for the study of an evolutionary dynamics with the aid of the probability distributions of paths. We carry out the study in well-mixed finite populations with fixed size *N*. There are two states (the ancestral population), cooperation (*i* = 1) and defection (*i* = 2). The number of cooperatiors is denoted by $m$ and updated by the Moran process^24^. Fitness is simply given from the expected payoff. We define the birth–death process given by the prisoner's dilemma with the payoff matrix ($c>a>d>b$):

$$\begin{array}{cc}& \begin{array}{cc}C& D\end{array}\\ \begin{array}{c}C\\ D\end{array}& \left(\begin{array}{cc}a& b\\ c& d\end{array}\right)\end{array}.$$

Then, the expected payoff for *i* = 1 and 2 individuals are, respectively, given by

6 $$\begin{aligned} {F}_{1}&=\frac{a\left(m-1\right)+b(N-m)}{N-1},\\ {F}_{2}&=\frac{cm+d(N-m-1)}{N-1}. \end{aligned}$$

**Figure 5:**
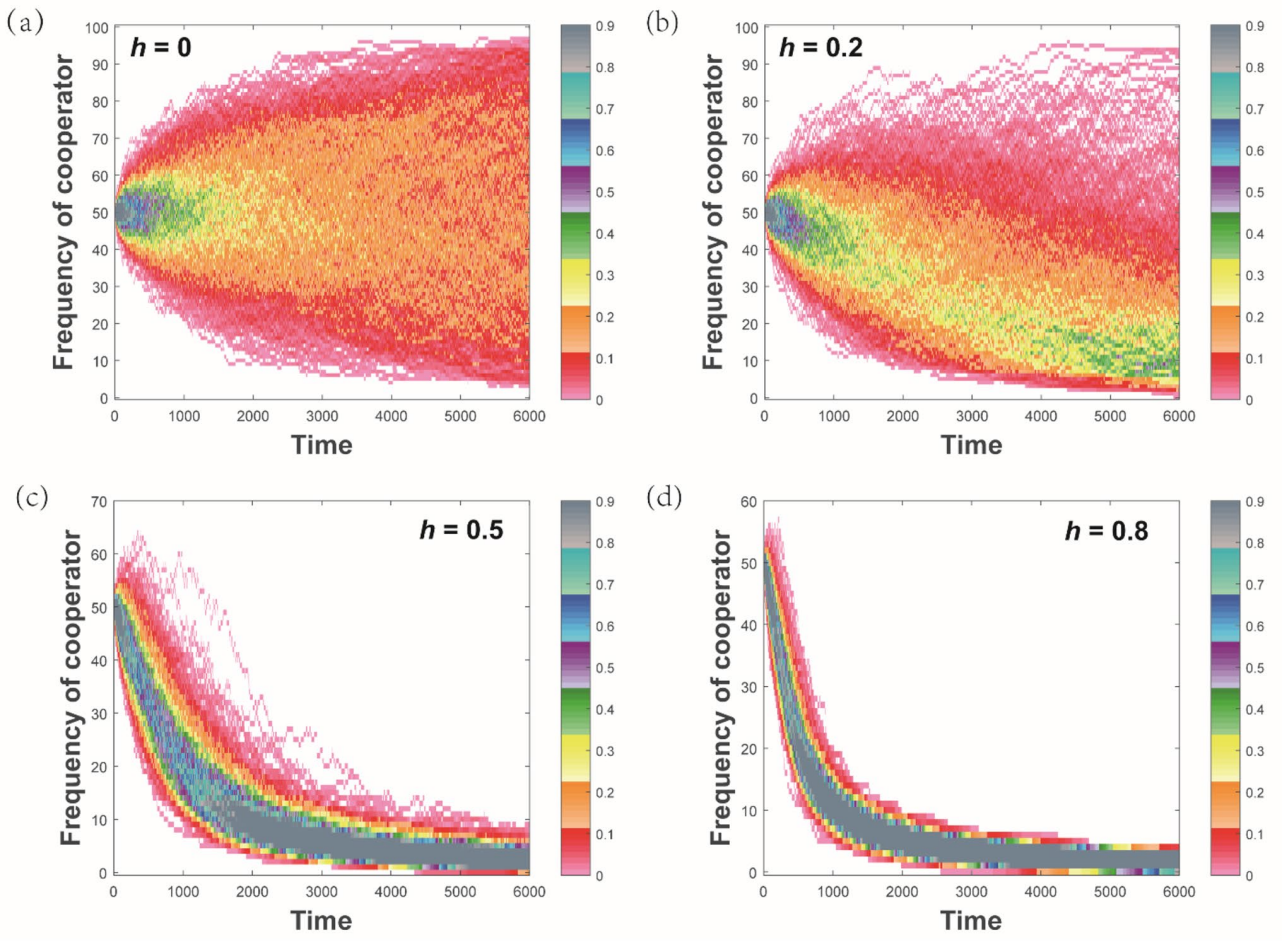
The distributions of the evaluation path probability under different selection pressure in the simulation of evolutionary game. (**a**–**d**) correspond to different selective pressure, *h* = 0, 0.2, 0.5, 0.8, respectively.

In a finite population *N*, the fitness of the two strategies is frequency dependent. We also consider the selection intensity parameter $h$ ($0<h<1$). The corresponding fitness at each step is then ${w}_{\mathrm{1,2}}=1-h+h{F}_{\mathrm{1,2}}$. This process has two “absorbing states” $m=0$ and $m=N$: once the process has reached such a state, it will stay there forever. We investigate the trajectory from the initial state $m=N/2$ during 6000 simulation steps. In order to seek the pathways most likely to appear, we simulate 5000 evolutionary processes and analyzed the probability distribution of evolution trajectories by Monte Carlo. An evolutionary path can be described by a series of frequencies at successive history steps. In Fig. 5, we show the distribution of pathways under four levels of selection pressure.

From the simulation, one can see that the trajectory with maximum probability is located at the middle area of the curve. Although all the paths start from the same initial state and evolve to the same final state, they have different probabilities. If the selection pressure is not too strict (weak selection), the expected payoff ${F}_{\mathrm{1,2}}$ is multi-value at each update step. The evolutionary path recured by the Price’s rule is therefore not unique (In our simulation, the price’s formula corresponds to the update rule). Then, a set of evolutionary paths could produce the same absorbing states. The pathways most likely appeared in the evolutionary process locate at the middle area and might not be derived by absorbing to the direction of equilibrium state *m* = *N*. That is exactly an exhibition of what the selection preference of the path-dependent selection is not completely consistent with the Price’s description.

We consider different selection intensities, assuming that the species evolution is influenced by one or more factors (e.g., the interaction of different phenotypes, environment fluctuation), the parameter *h* being assigned ranging from 0  to  1. Small *h* means that there has low selection preference between cooperators and defectors at each step. For *h* = 0, all the evolutionary paths are produced with equal probability and selection is almost neutral. It is only constrained the initial value. With increasing selection pressure *h*, the distribution of paths that the strategy space cross would be more concentrated. For *h* = 0.8, only a few evolutionary paths are permitted and randomness decreases gradually. Whereas for an infinite great selection pressure, evaluation is allowed in a single path and the selection becomes directional.

## Discussion

Evolutionary biology assumes that natural selection has already maximized fitness, in which we observe in nature is close to the trait with a fitness-maximizing equilibrium or the evolutionarily stable strategy^22^. Underlying this view is the assumption that the organism we observe is the final state of a long evolutionary process over many generations^22^. However, if there exist feedbacks between the adaptation and environments, such as the case of the finite population, the intermediate generations are a worthy addition to the evolution^28,31^. For the intermediate process of the speciation which we focus, evolutionary trajectory emphasizes that selection acts upon the probability distributions of paths rather than the phenotypes of the given equilibrium point^31^. Therefore, the evolutionary path probability parameter ${\mathrm{P}}_{\mathrm{path}}$ is suitable for a sequence of evolutionary states and a more feasible patterns in the inquiry of the selection rule of the evolutionary paths.

In our framework, the evolutionary path contains the information of the junctions between the two repetitions which represent the change of fitness parameter. As mentioned by Wright, in a frequency-dependent selection the average fitness need not grow continually^39^. These junctions are thus informative and alter the fitness parameter^5,40^. Especially, evolutionary changing toward equilibria occurs slowly in the case of weak selection pressure (Fig. 5a,b). If the new ecological equilibria are generated before the population structure reaches ecological equilibria, the varying fitness makes the evolution not always stay at the maximum point. Some lower fitness traits may thus appear in the evolutionary paths with lower probability. Unless we assume that population dynamics are typically considered operating at a much faster pace relative to evolutionary processes (ignoring effects of population dynamics on the fitness, such as infinite population size)^41^, the junctions could not be dropped out.

In the meaning of the evolutionary paths, if the selection pressures are weak, the traits with low pay-offs might not be out-competed promptly in the intermediate process (Fig. 5). Thus, even an evolution starting from one initial state and falling into the same final state would allow for multiple intermediate paths. In fact, several experiments have been found including multiple different paths^21,39,42^ and the multiple path evaluation will always occur in the substitution in individual base pairs of DNA^42^. In such a pattern, some possible evolutionary chains might develop along with lower reproductive rates even if natural selection favors the trait with increasing fitness at each evolutionary step. When the evolution is multiple-pathway, it means that the expected fitness is the same but the same intermediate state is not exactly equal, i.e., sympatric species, may exist.

Since the distribution of evolutionary path probability ${\mathrm{P}}_{\mathrm{path}}$ is a multi-dimensional function taking the frequency ${q}_{i}^{(t)}$ as the dependent variables at the whole evolutionary step, it will extend the conception of species to speciation. This description makes the essence of any evolution to a process where the populations will not only keep their divergence upon contact but also be able to continue to diverge^43,44^. It contains a unification of the statics and dynamic properties in an evolutionary process. We should stress that the stationary population distribution ${q}_{i}^{({t}_{n})}$ at a given time is equivalent to the evolutionary path probability ${\mathrm{P}}_{\mathrm{path}}$ only in one small step or a fitness stationary case. Specifically, if the selection pressure is small, all evolutionary paths occur in a nearly identical probability (Fig. 5a). With the increase of selection pressure, the distribution of the paths is more concentrated and the direction of the evolutionary path shows up gradually. If the selection pressure is strong enough, evaluation is allowed in a single path and the becomes directional, i.e., the traditional Price equation^13^.

In conclusion, we focus on the selection rule based on the path-depended. Stating from the path-depended selection, we found that the evolutionary path expands several missing but meaningful phenomena in the fitness-based theory. Drawing on statistic physics, we suggest that the conception of the probability distribution function (or rather functional) could be introduced to identify the evolutionary path. Considering the evolutionary path, some individuals with lower fitness could coexist with others due to they get a large evolutionary path probability ${\mathrm{P}}_{\mathrm{path}}$ if selective pressure of the natural selection is not too strong. The distinction selective mechanism of the evolutionary path probability in the whole evolutionary process might seed a potential to explain the diversified fitness evolutionary strategies (e.g., coexistence of altruism and selfishness) and thus species diversity.

## Method

We could give a mathematical formalism based on the probability function of evolution trajectories. In general, the growth value can be written as

7 $$f\left(x,t\right)+g\left(x,t\right)\xi \left(t\right),$$

where $f\left(x,t\right)$ and $g\left(x,t\right)$ are smooth functions, $f\left(x,t\right)$ involved fitness (received feedback from other generations), and $\xi \left(t\right)$ is the term for environmental fluctuations. We consider an evolution process.

8 $$\dot{x}=f\left(x,t\right)+g\left(x,t\right)\xi \left(t\right)$$

If the phenotype began with ${x}_{0}$ at ${t}_{0}$, taking account of the path integral, we obtain probability of evolutionary when the final phenotype is *x* at *t*

$$P\left(x,t;{x}_{0},{t}_{0}\right)=\int D[\mathrm{x}(\mathrm{t})]\mathrm{exp}\left[-\frac{1}{h}{\int }_{{t}_{0}}^{t}\mathrm{d}\tau L[x\left(t\right), \dot{x}\left(t\right)\right],$$

with $L\left[x\left(t\right),\dot{x}\left(t\right)\right]$ being the so called Lagrangian in Lagrangian mechanics analysis. $L\left[x\left(t\right),\dot{x}\left(t\right)\right]$ is another form of the Eq. (8). The probability densities of each trajectory are, following Ref.^45^,

$$P\left[\mathrm{x}\left(\mathrm{t}\right)\right]=D\left[\mathrm{x}\left(\mathrm{t}\right)\right]\mathrm{ exp}\left[-\frac{1}{h}{\int}_{{t}_{0}}^{t}\mathrm{d}\tau L[x\left(t\right), \dot{x}\left(t\right)\right].$$

It seems that this is a complex formula, but still gives a general mathematic formula and practical execution for study of the distribution of an evolutionary trajectory. In fact, the evolutionary significances of the path probability would become clearly in a specific system as our contrived example of evolutionary game simulation.

## Data Availability

This work is pure theoretical research and there is no experimental data. All data generated or analyzed during this study are included in this published article. The programs for the current study are available from the corresponding author on reasonable request.
