# Author Correction: Path probability selection in nature and path integral

**DOI:** 10.1038/s41598-022-27241-4

**Published:** 2022-12-30

**Authors:** Chao Wang, Min-Lan Li, Rui-Wu Wang

**Affiliations:** 1 School of Ecology and Environment, Northwestern Polytechnical University, Xi’an, 710072 Shaanxi People’s Republic of China; 2 School of Mathematics and Statistics, Northwestern Polytechnical University, Xi’an, 710072 Shaanxi People’s Republic of China

Correction to: *Scientific Reports* https://doi.org/10.1038/s41598-022-20235-2, published online 09 November 2022

The original version of this Article contained errors in the Abstract.

“However, this basic idea of biological evolution, which is mathematically described by Price equation or its relations, has not fully considered feedback effects from the environment or other generations.”

now reads:

“However, this basic idea of biological evolution, which is mathematically described by Price equation or its related models, has not fully considered feedback effects from the environment or other generations.”

Additionally,

“This mechanism could potentially explain fitness evolutionary strategies with the diversified fitness (e.g., coexistence of altruism and selfishness) and thus species diversity.”

now reads:

“This mechanism could potentially explain evolutionary strategies with the diversified fitness (e.g., coexistence of altruism and selfishness) and thus species diversity.”

The original Article has been corrected.

